# Improved ethanol yield and reduced minimum ethanol selling price (MESP) by modifying low severity dilute acid pretreatment with deacetylation and mechanical refining: 2) Techno-economic analysis

**DOI:** 10.1186/1754-6834-5-69

**Published:** 2012-09-11

**Authors:** Ling Tao, Xiaowen Chen, Andy Aden, Eric Kuhn, Michael E Himmel, Melvin Tucker, Mary Ann A Franden, Min Zhang, David K Johnson, Nancy Dowe, Richard T Elander

**Affiliations:** 1 National Bioenergy Center, National Renewable Energy Lab, 15013 Denver West Parkway, Golden, CO, 80401, USA

**Keywords:** Techno-economic analysis, Biofuel, Integrated process, Pretreatment, Mechanical refining, and Deacetylation

## Abstract

**Background:**

Our companion paper discussed the yield benefits achieved by integrating deacetylation, mechanical refining, and washing with low acid and low temperature pretreatment. To evaluate the impact of the modified process on the economic feasibility, a techno-economic analysis (TEA) was performed based on the experimental data presented in the companion paper.

**Results:**

The cost benefits of dilute acid pretreatment technology combined with the process alternatives of deacetylation, mechanical refining, and pretreated solids washing were evaluated using cost benefit analysis within a conceptual modeling framework. Control cases were pretreated at much lower acid loadings and temperatures than used those in the NREL 2011 design case, resulting in much lower annual ethanol production. Therefore, the minimum ethanol selling prices (MESP) of the control cases were $0.41-$0.77 higher than the $2.15/gallon MESP of the design case. This increment is highly dependent on the carbohydrate content in the corn stover. However, if pretreatment was employed with either deacetylation or mechanical refining, the MESPs were reduced by $0.23-$0.30/gallon. Combing both steps could lower the MESP further by $0.44 ~ $0.54. Washing of the pretreated solids could also greatly improve the final ethanol yields. However, the large capital cost of the solid–liquid separation unit negatively influences the process economics. Finally, sensitivity analysis was performed to study the effect of the cost of the pretreatment reactor and the energy input for mechanical refining. A 50% cost reduction in the pretreatment reactor cost reduced the MESP of the entire conversion process by $0.11-$0.14/gallon, while a 10-fold increase in energy input for mechanical refining will increase the MESP by $0.07/gallon.

**Conclusion:**

Deacetylation and mechanical refining process options combined with low acid, low severity pretreatments show improvements in ethanol yields and calculated MESP for cellulosic ethanol production.

## Background

In order to alleviate the “oil addiction” and “oil crisis” of the United States, the U.S. Department of Energy has set up goals to produce 36 billion gallons of biofuel per year on a Btu-adjusted basis by 2022, which will replace 30% of the 2004 U.S. motor gasoline demand [1]. In addition to the biofuel production target, biofuel development is facing two other important targets: cost and sustainability. EIA projects the West Texas Intermediate (WTI) crude oil spot price to average about $88 per barrel over the second half of 2012 and the U.S. refiner acquisition cost (RAC) of crude oil to average $93 per barrel [2]. In a recent techno-economic analysis report published by the National Renewable Energy Laboratory (NREL), the current target minimum ethanol selling price (targeting MESP) for cellulosic ethanol process is $2.15/ gallon [3]. Producing biofuel economically is equally important to achieving the production and sustainability targets.

Pretreatment is one of the important steps in the biological conversion of biomass feedstock to bioethanol. As the first step in the process, pretreatment plays a critical role in preparing biomass for enzymatic conversion to C5 and C6 sugars and, in some processes, directly hydrolyzing a portion of structural carbohydrates to oligomeric and monomeric sugars [4]. The pretreatment step has been projected to be one of the most expensive capital investments in the biochemical conversion process, regardless of the technologies used, in various studies [5-7]. More important, it has significant impacts on the downstream conversion steps, such as enzymatic hydrolysis and fermentation, by pretreated biomass as well as all the chemicals introduced in the pretreatment.

In the current state of technology reported by NREL [3], dilute acid pretreatment is used at relatively mild conditions. A downstream oligomer hold reactor may be needed to further convert the residual xylo-oligomers to xylose monomers at a temperature of 130°C, which is lower than the pretreatment reaction temperature of 150-190°C. This process was found to convert 80% of the xylan to soluble xylose monomer with 6% loss to furfural [7]. Approximately 9% of the soluble oligomeric sugars were solubilized, and approximately 5% of the xylan was left in the insoluble solids. The current state of pretreatment technology brings many technical problems and process issues [8]. As discussed in our companion paper, we have developed a low-severity dilute acid pretreatment method combined with deacetylation, mechanical refining, or hydrolysate solids washing to solve these problems while maintaining or improving ethanol yields [8].

However, applying these process options of deacetylation, mechanical refining, and solids washing to a biochemical conversion ethanol process could potentially introduce high capital investment to the overall production cost. Deacetylation brings in extra chemical costs such as sodium hydroxide (NaOH) and extra capital such as solid–liquid separation. Mechanical refining requires investment in additional equipment and operating costs for power consumption. Solids washing after dilute acid pretreatment will contribute significantly to capital costs for solid–liquid separation equipment. Therefore, a techno-economic analysis (TEA) is required to compare the cost benefit of all of these options.

In this study, a TEA based on NREL’s AspenPlus model was applied to compare the MESP of ethanol produced from two varieties of corn stover under base (control) and modified process cases. Pretreatment process alternatives with the options of deacetylation, mechanical refining, and a solids washing step are investigated. In this paper the economic impact of introducing deacetylation, mechanical refining, and washing are discussed, as an important addition to the companion paper [8].

## Results and discussion

### Corn Stover varieties

Compositional analysis of three types of native corn stover is shown in Table 1, including corn stover used in the NREL 2011 design report [3], and two types of corn stover harvested from the Kramer farm in Wray, Colorado. The three corn stover varieties have similar compositions, containing 34%-35% glucan, 19%-23% xylan, 12%-16% lignin, and about 2%-3% acetyl groups. The three corn stover varieties are termed as corn stover composition used in the NREL design report as 2011 design, Kramer 34M95 and Kramer 33B51 in the work. The stover appeared to contain sizable amounts of soil and other contaminants. 

**Table 1:** Compositional analysis of native corn stover species studied; bulk moisture is 20 wt% for all three feedstocks

**(dry wt%)**	**2011 Design**	**Kramer 34M95**	**Kramer 33B51**
Glucan	35	34	34
Xylan	20	23	19
Lignin	16	13	12
Ash	5	3	5
Acetate	2	3	3
Protein	3	3	3
Extractives	15	10	15
Arabinan	2	3	3
Galactan	1	1	1
Mannan	1	0	0
Sucrose	1	6	6
Total structural carbohydrate	59	61	57

**Figure 1:**
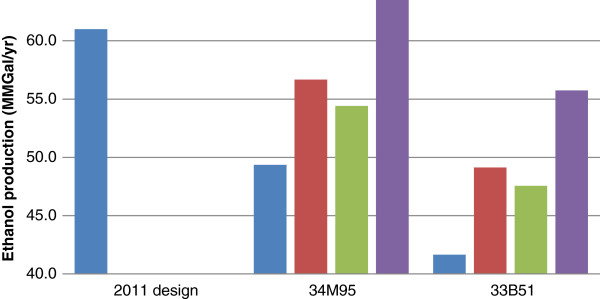
**Calculated annual ethanol production (million gallons).** (blue bar) Control; (red bar) Deacetylation; (green bar) Refining; (violet bar) Deacetylation + Refining.

Some variations have been observed for sucrose content because it is highly dependent on harvesting, handling, and storage. The total structural carbohydrate content of the corn stover composition in the NREL 2011 design report is 59%. This value is about 2% lower that of than Kramer 34M95 and 2% higher than that of Kramer 33B51. The total structural carbohydrate content in the feedstock impacts the sugar yield as well as ethanol yield. As illustrated in Figure 1, the annual ethanol production is determined by annual ethanol yield based on a constant annual feedstock feeding rate.

**Figure 2:**
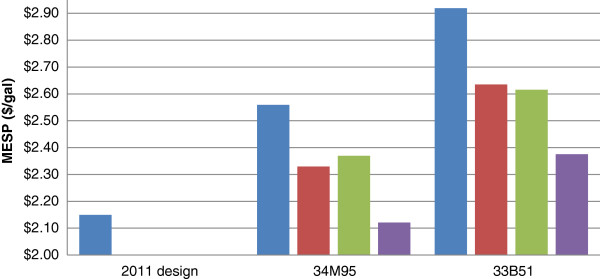
**MESP results of five feedstock types with and without deacetylation, with and without mechanical refining.** MESP results with options of deacetylation and mechanical refining. (blue bar) Control; (red bar) Deacetylation; (green bar) Refining; (violet bar) Deacetylation + Refining.

Due to the milder pretreatment conditions applied (shown in Table 2), the ethanol yield for both feedstocks studied here is lower than the yield achieved in the 2011 NREL design case, resulting in a reduction in ethanol annual production, shown in Figure 1, and an increase in MESP (over $0.40), shown in Figure 2. The purpose and benefits of applying low acid pretreatment was discussed in detail in the companion paper [8]. 

**Table 2:** Low acid and low temperature pretreatment conditions used in this study, compared with conditions from the 2011 NREL design model

	**2011 Design**	**Low Acid in this Study**
Sulfuric acid loading	22 mg/g dry biomass	8 mg/g dry biomass
Residence time	5 minutes	20 minutes
Temperature	158°C	150°C
Pressure	5.5 atm	4 atm

A 30% total solid loading is applied for both conditions.

Applying a deacetylation step increases the pretreatment yields by improving xylose yields, leading to improved ethanol production per unit (ton) of biomass. Figure 1 illustrates the comparison between the control cases where feedstock variability and differences in recalcitrance affect the annual ethanol production of a facility of 2,000 metric dry ton (MT)per day corn stover. In addition, mechanical refining improves the ethanol production from pretreated slurries (native, non-deacetylated) after mechanical refining have improved annual ethanol production due to increased enzymatic hydrolysis yields, although not as much as for deacetylation. Combining both deacetylation and mechanical refining into the process design improves the annual ethanol production from 49 to 64 MM gallon/yr for 34M95 corn stover, and from 42 to 56 MM gallon/yr for 33B51 corn stover, as shown in Figure 1.

### Impact of deacetylation on cost

Overall economics are still dominated by the conversion costs (non-feedstock costs portion). Therefore, improving the ethanol yields should improve the cost (i.e., the MESPs). The MESPs calculated for the two feedstock harvests are reduced with incorporation of a deacetylation step. Compared with the control cases, pretreatment with deacetylation decreases MESP by $0.23 and $0.29 per gallon, respectively, for feedstock varieties 34M95 and 33B51, as shown in Figure 2.

### Impact of mechanical refining on cost

Mechanical refining also improves the process economics (see Figure 2), even when increased capital investments and power consumption are taken into consideration. Compared with control cases, pretreatment with mechanical refining reduces MESP by $0.19 and $0.30 per gallon, respectively, for feedstock varieties 34 M95 and 33B51, as shown in Figure 2. The combination of deacetylation and mechanical refining shows further cost reductions than found using either process option alone (Figure 2). Total MESP reductions are $0.44 per gallon for corn stover 34M95 and $0.54 per gallon for 33B51. Note that for 34M95 corn stover, a combination of deacetylation and disk refining process options yielded 64 MM gallons ethanol production per year compared to 49 MM gallons in the control case, representing a 25% yield improvement. The MESP calculated for this scenario, $2.12 per gallon, approaches the value of $2.15 per gallon that is reported in the 2011 NREL design case.

### Impact of pretreated solids washing on cost

**Figure 3:**
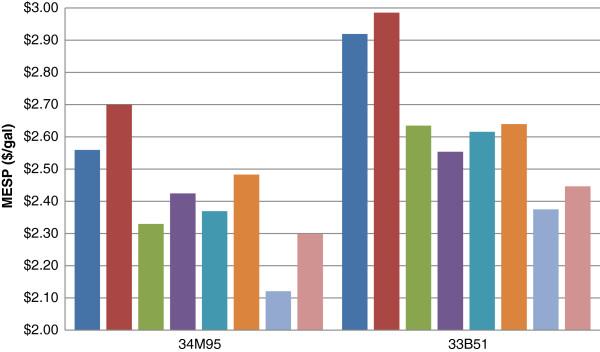
**MESP comparison of whole slurry and washed solids cases.** (blue bar) Control; (red bar) Control + wash; (green bar) Deacetylation; (violet bar) Deacetylation + wash; (acqua blue bar) Refining; (orange bar) Refining + wash; (sky blue bar) Deacetylation + refining; (pink bar) Deacetylation + refining + wash.

Ammonia conditioning has replaced over-lime conditioning of pretreated hydrolysate slurries, allowing the conditioned hydrolysate slurries to be enzymatic hydrolysis-friendly with non-washed solids. In the non-washed solids process option, the solid–liquid separation unit following pretreatment is eliminated, resulting in lower capital and operating costs [3], as well as lower waste streams [9]. However, whole slurry enzymatic saccharification suffers severely from glucose and xylose yield losses because the high concentrations of xylose in the pretreated slurry inhibit the xylanase enzyme activity in commercially available cellulase and hemicellulase enzymes. The inhibition decreases the conversion of xylan to xylose, which therefore decreases cellulase enzyme accessibility to the cellulose microfibrils in the pretreated substrates, resulting in lower cellulose-to-glucose yields. The addition of a solids washing step significantly reduces this inhibition and increases overall sugar yields. However, the washed solids option is strongly penalized with high capital costs of the solid–liquid separation unit. The amount of total project investment (TPI) increases with different corn stover varieties by roughly $130 MM, only varying slightly on other pretreatment process options. The variation of capital cost increments is mainly due to the scale of the solid–liquid separation step, while the base capital assumption is kept the same for all the cases. The additional cost of capital in the solid–liquid separation step cannot be offset by improved ethanol yield. As a result, most of the cases with solid–liquid separation will have higher MESPs, as shown in Figure 3. 

Since the solid–liquid separation unit alone contributes significantly to the total project investment, reducing uncertainties in the capital and operating costs of installing the solid–liquid separator should be further addressed. Additional work is proposed to characterize options for filtering pretreated slurry solids and to contact vendors for quotes on suitable solid–liquid separation equipment.

### Sensitivity analysis of pretreatment capital costs

The capital cost of the pretreatment reactor is a significant contributor to MESP. Lowering the pretreatment temperature to 150°C or less and the acid concentration to 0.5 wt% sulfuric acid or less allows the use of lower-cost stainless steel as the construction material for the reactor. Expensive Hastelloy 2000 has been used where the temperature in the corresponding reaction zone is over 150°C, as currently is done at NREL in the pilot-scale Metso horizontal reactor. The pretreatment reactor has an assumed $30 MM purchased cost per unit due to the exotic material of construction [3]. Stainless steel grade 904 L or similar duplex stainless steel could potentially be used as the building material for a pretreatment reactor. However, coupon corrosion tests of 904 L and other duplex stainless steels using corn stover hydrolysates to test the effects of corrosion and erosion are still needed for reactor vendors to provide accurate quotations. Additionally, as discussed in our companion paper [8], less severe pretreatment does not require precise residence time control. Instead of expensive horizontal screw pretreatment reactors, vertical reactors with simple configurations could potentially further reduce the capital investment of the pretreatment reactor. In this study, although results from a reduction of capital investment for the pretreatment reactor are not available, a sensitivity study has been performed. In this sensitivity analysis, the reactor cost was reduced by assuming a 50% reduction in pretreatment reactor cost if stainless steel could be used. The MESP increases linearly from $2.01 to $2.12 per gallon of ethanol for corn stover 34M95 if the pretreatment reactor costs increase from $15 MM to $30 MM. Similar results were found for the other varieties of corn stover feedstock studied in this work. The reductions in MESP are of the order of $0.11-$0.14 per gallon of ethanol, simply due to an average reduction of total project investment (TPI) of 50% on pretreatment reactors.

### Sensitivity analysis for energy consumption of mechanical refining on cost

Mechanical refining of pretreated corn stover slurries significantly increases the overall digestibility of the pretreated corn stover, as shown by improved sugar yields in the companion paper [8]. The mechanical refining treatment loosens the structure of the fiber within the cell wall (internal fibrillation) in addition to creating external fibrillation that increases cellulose microfibril exposure to cellulase enzymes. The increased fibrillation caused by the high shearing forces of mechanical refining can significantly increase surface area, which leads to increased enzyme accessibility [10]. However, the incorporation of a mechanical refining process option has long been considered uneconomical because of its high energy consumption. Recent literature reports have stated that the energy consumption for mechanical refining is less than 20 kWh/dry ton biomass using spruce [10,11]. Little research has been reported about the energy consumption of mechanically refining corn stover, and the effect on the MESP of incorporating mechanical refining into a biochemical process has not been systematically studied. In this study, we performed a cost sensitivity analysis of the MESP by incorporating a mechanical refining process option into the existing model and varying the energy consumption. We found that MESP increases by $0.07 per gallon of ethanol if energy consumption increases from 19 to 200 kWh/dry ton, and it increases by $0.15 per gallon of ethanol if energy consumption increases to 400 kWh/dry ton.

### Uncertainty analysis of the mechanical refiner capital costs

The MESP is shown to vary with different assumptions for capital costs of the mechanical refining process option. For disk refiners with lower power consumption (less than 50 kWh/dry ton biomass), a $2 MM direct capital cost was used. However if higher power consumption (greater than 150 kWh/dry ton biomass) is required to improve enzyme digestibility, the capital could reach $10MM because additional disk refiners will be needed in order to achieve the desired refining effect and biomass throughput. In this study, we used $2MM per disk refiner unit (one extra unit is needed for backup) direct capital investment because the literature reports power consumption to be less than 20 kWh/dry ton biomass [10,12]. If higher power requirements are needed, then the direct capital costs of the mechanical refining process option increase to $10MM, and an additional $0.04 would be added to MESP for capital.

## Conclusion

A series of techno-economic analyses were performed for pretreatment processing technologies using low acid, low severity process options to increase the overall pretreatment, enzymatic saccharification, and fermentation yields. Results from bench-scale experiments for corn stover compositions and sugar and ethanol yields were applied as well as compared to the current 2011 NREL design base case model, and simulations were run using the various combinations of process options. The MESP for bioethanol produced from corn stover was estimated for incorporation of the following process options:

Different corn stover varieties with different levels of recalcitrance

Control cases using low severity pretreatment

Deacetylation of the corn stover feedstocks prior to pretreatment, with or without washing

Mechanical refining prior to enzymatic hydrolysis, with or without washing following pretreatment and prior to enzymatic hydrolysis.

Bench-scale experiments showed that deacetylation, mechanical refining, and hydrolysate solids washing all improved the final sugar and ethanol yields for the low severity pretreatment conditions used in this study. In general, the calculated MESP decreased from high numbers ($2.92/gallon for the low severity pretreatment control case) when the various process options were incorporated, with the exception of the washing option. Because of the expense of the solid–liquid separation step, the washing process option showed MESP increases of up to $0.15 per gallon of bioethanol produced, even when both the enzymatic saccharification yields of glucose and xylose and the fermentation yields increased substantially by decreasing the inhibitor levels. Deacetylation not only improved xylose monomer yield during pretreatment but also increased the digestibility of residual xylan in the low severity pretreated corn stover feedstocks studied. By removing acetyl groups prior to pretreatment, less acetic acid is released into the downstream process, leading to lower amounts of neutralizing chemicals added during enzymatic saccharification and higher ethanol yields in fermentation. Mechanical refining benefits the enzymatic saccharification significantly. By applying high shearing force, the pretreated corn stover was made more digestible due to multiple property changes in the cellulose structure. Washing solids is known to be expensive because of the additional cost of solid–liquid separation equipment. However, washing also overcomes the inhibition of hemicellulase brought on by high xylose concentration in hydrolysates, resulting in further conversion of unreacted xylan in the solids. By combining two of the above options or all three of them, higher sugar/ethanol yields were indeed achieved.

The MESP of corn stover ethanol produced by current process technology is significantly driven by the final ethanol yield. For Pioneer 34M95 corn stover, deacetylation increased the annual ethanol production by about 8 million gallons, and correspondingly the MESP of ethanol decreased by about $0.23 per gallon. For Pioneer 33B51 corn stover, deacetylation increased the annual ethanol production by about 7 million gallons and lowered the MESP by about $0.29 per gallon of ethanol. Similar to deacetylation, mechanical refining decreased the MESP mainly by improving ethanol yields. Also, combining these two options increased the ethanol yield significantly and lowered the MESP, offsetting the combined cost increases for capital and operating costs. If a less expensive reactor can be utilized in these lower severity pretreatments, it may be possible to lower the MESP by an additional $0.11 to $0.14 per gallon of ethanol if a 50% reactor cost reduction is assumed. Washing of the solids was shown to improve ethanol yields for all cases studied; however, large increases in the calculated MESP were found due to the high capital costs of solid–liquid separation equipment. An increase in MESP of $0.07 per gallon of ethanol was calculated if the energy requirements for disk refining were increased 10-fold.

### Future research

There are still many uncertainties in the current research that need further investigation. More accurate quotations on industrial scale equipment, more accurate energy consumption measurements on mechanical refining, accurate sugar and ethanol yield data from pilot-scale experiments, and more accurate solid–liquid washing equipment costs will improve the MESP estimates. In the future, optimization of deacetylation and mechanical refining process options could improve the economic feasibility of using low acid, low severity pretreatment process options.

## Methods

### Conceptual Process Design

**Figure 4:**
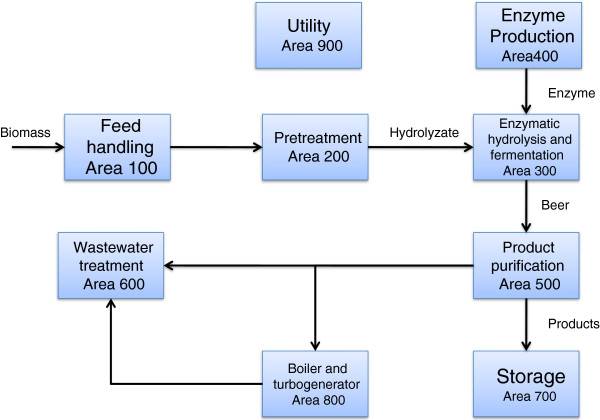
Simplified flow diagram of the overall process.

The process design includes feedstock handling and storage, product purification, wastewater treatment, lignin combustion, product storage, and all other required utilities. In all, the process is divided into nine areas using the NREL 2011 biochemical design report as the basis, as shown in Figure 4.

We chose a plant size of 2,000 MT per day, with 8,406 annual operating hours. The feedstock, in this case milled corn stover, is delivered to the feed handling area (Area 100). Only minimum storage and feed handling are required in the current biochemical conversion process. The feedstock is treated with acid in pretreatment (Area 200) to liberate the hemicellulose sugars to break down the biomass and is then washed and or neutralized for enzymatic hydrolysis. Enzymatic hydrolysis (Area 300) is initiated in a high-solids continuous reactor using enzyme addition. The partially hydrolyzed slurry is next batched to a system of parallel anaerobic bioreactors. Hydrolysis is completed in the batch reactor, and then the slurry is cooled and inoculated with an engineered, xylose-fermenting organism *Zymomonas mobilis* for fermentation. After 5 days of enzymatic hydrolysis and fermentation, most of the cellulose and xylose will have been converted to ethanol. The resulting beer is sent to the product recovery train (Area 500). Oligomer sugars are not considered fermentable in the base case. The beer is separated into ethanol, water, and residual solids by distillation and solid–liquid separation. Ethanol is distilled to a nearly azeotropic mixture with water then purified to 99.5% using vapor-phase molecular sieve adsorption. Solids from the distillation bottoms are separated and sent to the combustor (Area 800) while the liquid is sent to wastewater treatment (Area 600). Onsite utility integration (Area 900) is included for cooling water system, chilled water system, process water manifold, and power systems.

### Pretreatment

**Figure 5:**
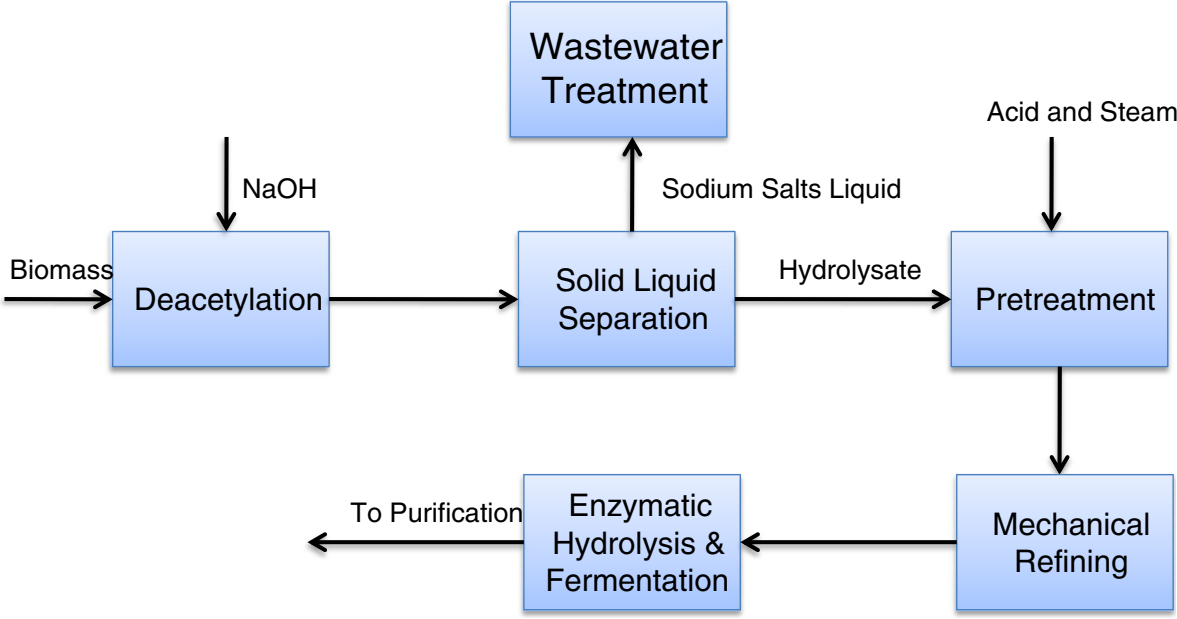
Schematic process flow diagram using process options of deacetylation prior to pretreatment and mechanical refining after pretreatment.

In this study, the pretreatment is carried out in a bench-scale steam explosion reactor. If deacetylation and mechanical refining are applied to further improve the process design in the pretreatment (Area 200), biomass feedstock starts with deacetylation, followed by solid–liquid separation, then dilute acid pretreatment and enzymatic hydrolysis. Mechanical refining of pretreated solids is utilized before enzymatic hydrolysis but after washing if hydrolysate solids washing is applied, as shown in Figure 5. The pretreatment reaction conditions are milder than what has been modeled in the 2011 NREL design model, as compared in Table 2. In the control cases modeled with bench-scale data from different varieties of corn stover feedstock, a higher proportion of xylose oligomers and a lower proportion of degradation products resulted from the low severity pretreatments. The hydrolysate slurry is flash-cooled following pretreatment, which vaporizes a large amount of water, along with some of the acetic acid, furfural, and hydroxymethyl furfural (HMF). The flash vapor is then sent to the wastewater treatment area. There is no oligomer hold step in this design, so overall the acid loading (8 mg/g dry biomass) is much lower than what has been modeled in the 2011 NREL design model (22 mg/g dry biomass), shown in Table 2. The direct advantages of using the lower acid loadings are 1) lower severity of pretreatment reaction conditions, 2) lower requirements for ammonia in the neutralization and conditioning step, and 3) longer residence time that might enable a cheaper vertical design. Between these considerations and the potential consideration for less costly material of construction due to lower corrosion potential, the 50% case for lower pretreatment capital cost is defendable. For a pretreatment reactor using Incoloy clad 825, a capital cost of $30 MM is assumed in the control cases.

### Deacetylation

Biomass feedstocks are mixed with caustic soda (NaOH) in the deacetylation reaction tank. The loading of NaOH is 0.04 g per gram dry biomass. The reaction is held at 80°C for one hour based on bench-scale studies. A solid–liquid separation unit is needed to remove the sodium acetate in the liquid stream. It was found that 75 wt% of the acetate was removed from the feedstock as sodium acetate based on bench-scale studies. Lignin loss is 20 wt% into the liquid stream and other solids losses are relatively minimal (less than 3%). The solid–liquid separation step can be performed using a pressure filter or belt filter, based on the relatively coarse particle size distribution of untreated corn stover (knife milled through a ½-inch rejection screen), as shown in Figure 5. The screw feeder of the Andritz design can also effectively remove moisture up to 60% total solids, therefore a separate solid–liquid separation unit has been removed by expanding the function of the design used in the 2011 NREL design case. Direct capital costs of the liquid separation unit are not known, so an estimated cost of $10 MM was used. This estimation is in between the costs of $35MM for the hydrolysate solid–liquid separation and $3.3MM for the lignin solid–liquid separation quoted by Outotec Larox filters [3]. Vendor quotations for the deacetylation solid–liquid filtration system are necessary in order to remove the cost analysis uncertainty caused by the direct capital estimation. The solids levels in the deacetylation step is assumed to be 30 wt% in order to avoid high water demand and high cost of wastewater treatment.

After the solid–liquid separation step, the liquid stream is sent to the wastewater treatment area in this process design, although sodium acetate can be recovered to regenerate sodium hydroxide for recycling. Please note that the recovery of NaOH requires significant capital investment. The deacetylated solids stream is sent to the dilute acid pretreatment reactor and mixed with sulfuric acid and steam to reach the pretreatment conditions shown in Table 2.

### Mechanical refining

**Figure 6:**
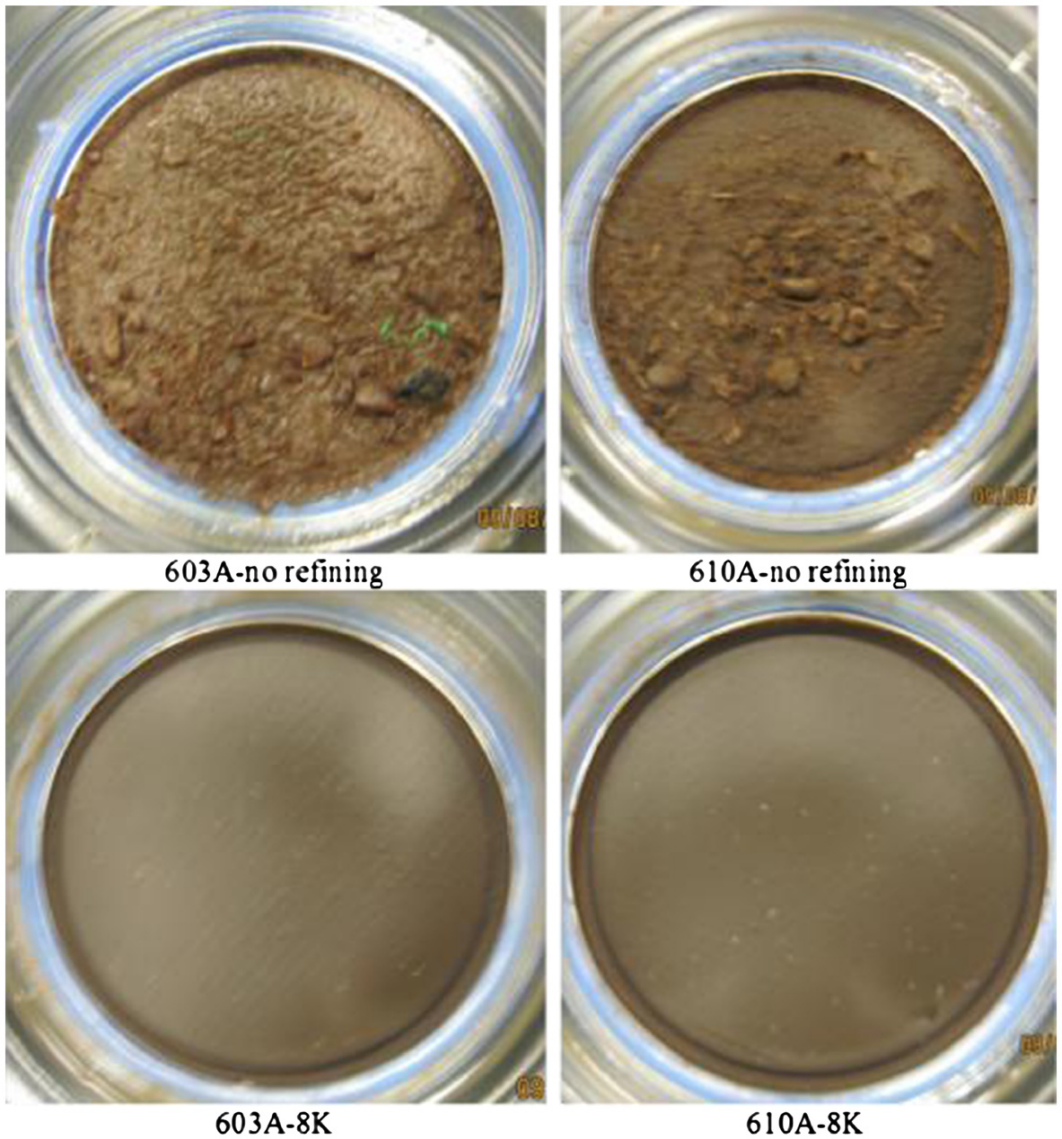
**Corn stover hydrolysate comparison between treatment without refining and refining using a total of 8,000 (indicated by 8 K) revolutions in a PFI refining mill.** Pioneer variety corn stover (33B51 and 34 M95) was pretreated at the low severity conditions of 150°C, 0.5 wt% H_2_SO_4_, for 20 min, diluted to 20 wt% insoluble solids, refined in a PFI mill for 8,000 revolutions, and enzymatically saccharified with 20 mg/g cellulose using Genencor GC220 cellulase enzyme preparation at 20 wt% total solids at 50°C, pH 5.0, 4 rpm for 168 h. The 168 h slurry was then filtered on 0.2 μ nylon filters.

The capital estimated for a double-disk refining unit is $2 MM per unit for this study. Typically for commercial-scale processes, a backup unit is needed to avoid maintenance downtime. Again, a vendor quotation is needed to narrow down the cost uncertainty introduced by incorporating refining technology. Energy consumption by this mechanical particle-size-reduction option was assumed to be 18.6 kWh/ton biomass (dry basis) [10], using 4,000 revolutions in a PFI refiner. It was found that increasing the total number of revolutions to greater than 8,000 did not further improve enzymatic hydrolysis yields. For a commercial-scale process, the power consumption could be lower than the bench-scale measurement of 18.6 kWh/ton dry biomass if only the tip speed of the rotation speed, instead of revolutions, is matched. The results of mechanical refining in a PFI refiner are shown in Figure 6, where the large particles normally found after low severity pretreatment are ground to a really fine particle consistency and then subjected to enzymatic saccharification. The lack of large particles after mechanical refining and enzymatic saccharification shows the effect refining has on increasing the enzymatic digestion yields, using a Genencor GC220 cellulase enzyme. The use of more advanced enzyme technology (e.g. Novozymes Cellic CTec2 and Cellic HTec2) would increase yields even further (up to 98% cellulose-to-glucose conversions were observed). 

### Solids washing

Washing solids has also been incorporated into the model as an alternative process option to the whole slurry enzymatic digestion of pretreated hydrolysate modeled in the 2011 NREL design model. In the whole slurry (no solids washing) option, the pretreated material is conditioned as a whole with ammonium hydroxide to neutralize acetic acid, sulfuric acid, and other organic acids, and to remove toxic inhibitors to the fermentative microorganism. In contrast, using the solids washing option, the pretreated material is diluted with recycled process water to a pumpable level and fed to the solid–liquid separation equipment. A Pneumapress pressure filter was used in the earlier 2002 NREL design model [5]; however, an Outotec Larox filters quotation of $35 MM per unit from the later 2011 NREL design report [3] is used in this study as the solid–liquid separator for separating solids from pretreated hydrolysate liquors. The washing step of dilution and separation of pretreated solids from liquors is performed prior to possible downstream mechanical refining. This not only allows a quicker and less expensive filtration of solids from low severity pretreated slurries, but also removes xylose inhibition of the xylanases in the enzyme cocktails prior to enzymatic saccharification so as to greatly improve xylan to monomeric xylose conversion yields. The liquor from filtration is neutralized with ammonium hydroxide. The final washed solids cake is conveyed off of the filter press onto a transport conveyer and into a slurry tank, where it is mixed with neutralized hydrolysate liquor and diluted to 20% total solids. Process and recycled water usage in this step is optimized to avoid high demands of fresh water. Only one washing cycle is assumed in this process option cost analysis in order to conserve capital investment costs and operating expenditures, in contrast to what was studied at the bench scale of more than one wash cycles were employed.

### Techno-economic analysis (TEA)

#### Capital and utility cost assumption

Techno-economic analysis (TEA) has been applied to this work. The material and energy balances and flow rate information for each process design option are generated using process simulation software packages. For these particular applications, Aspen Plus [13] was used. The updated biochemical cellulosic ethanol model [3] was used as a basis for modeling each process option, with supplied bench-scale experimental data for yields and operational conditions incorporated into the model. Raw material unit costs are cited from NREL’s 2011 design report [3], including caustic soda costs of $150 per dry ton. Utilities include steam (both low and medium pressure steam), power, water, and nitrogen gas. All costs are on a constant year-2007 dollars basis (see Table 3). 

**Table 3:** Raw material unit cost for cost analysis

**Raw material cost**	**Price ($2007)**
Corn stover	$58.50/bushel
Diammonium phosphate	$182.30/ton
Sulfuric acid	$32.10/dry ton
NH3	$300.00/ton
Caustic	$150.00/ton

Capital costs are developed mainly based on NREL’s 2011 design model. The same direct capital basis is used for the pretreatment reactor capital, but uncertainty introduced by pretreatment capital is also discussed. Capital for the deacetylation process option is taken mainly from existing NREL databases, except for the solid–liquid separator for the stream after deacetylation. A cost of $10 MM is assumed. For the mechanical refining process, a cost for the commercial particle size reduction equipment is not available, but in this report we assume a direct capital cost of $2MM. Talks with a vendor supplying mechanical refiners indicated that $2MM is within the range of commercial-scale 2,000 hp refiners without motors. Uncertainty in power usage and direct capital of the particle size reduction equipment (mechanical refiner) are also discussed in this work.

The scaling exponent for the power law was obtained from the NREL 2002 and 2011 design cases [3,5] for most of the equipment. For equipment not listed in the NREL design cases and for which we are not able to get vendor’s guidance, the exponent term is assumed 0.6. Standard NREL factors [3,5] were used to obtain the total project investment from the purchased equipment costs, factored to total project investment (TPI). The method for the discounted cash flow calculation in this study assumes 40% equity financing and 3 years construction plus 0.25 years start-up. The plant life is 30 years. The income tax is 35%. Working capital is 5% of fixed cost investment (FCI). The MESP is the minimum price that ethanol must sell for in order to generate a net present value (NPV) of zero for a 10% internal rate of return (IRR). This makes the MESP higher than a true cost of production.

It should be emphasized again that a certain percentage of uncertainty exists around conceptual cost estimates such as these. These values are best used in relative comparison against technological variations or process improvements. Use of absolute values without detailed understanding of the basis behind them can be misleading. Single factor sensitivity analysis is used in the study to capture effects on yields to address this issue.

In addition to process design changes for the deacetylation, mechanical refining, and hydrolysate solids washing options, several important cost assumptions that are different from the 2011 NREL design base case model are incorporated in the model and listed below:

All yields are based on results from bench-scale experiments from the companion paper [8]. Minor sugars reactions and yields are modeled using the reactions and yields of xylose if no data is available.

Liquid wastes from the deacetylation step are sent to the wastewater treatment area, assuming the anaerobic digester has no issues handling the extra amount of sodium from sodium acetate, instead of ammonia acetate.

No extra heat exchangers are used for the deacetylation reactor, because the reaction temperature is reached by dilution with preheated recycle water.

A mechanical refining step is applied after the pretreatment reactor in the whole slurry case, and is applied after the washing step in the washed solids option due to the complexity introduced by trying to filter fine particles generated in mechanical refining. The low severity pretreatment conditions preserve most of the corn stover feedstock's anatomical structure and morphology and allows for simple washing by filtration of the larger slurry particles. Refining after pretreatment followed by washing will require solid–liquid separation equipment that can handle very fine particles such as expensive centrifugation or Pneumapress-like equipment.

## Abbreviations

DOE: Department of Energy; EIA: Energy Information Administration; FCI: Fixed Cost Investment; Hp: Horse power; IRR: Internal Rate of Return; NREL: National Renewable Energy Laboratory; NPV: Net Present Value; MM: Million; OBP: Office of Biomass Program; RAC: Refiner Acquisition Cost; NaOH: Sodium hydroxide; TEA: Techno-economic Analysis; TPI: Total Project Investment; WTI: West Texas Intermediate.

## Competing interests

The authors declare that they have no competing interests.

## Authors’ contributions

LT designed conceptual process, studied TEA and drafted the manuscript. XC designed, conducted the experimental work including deacetylation, pretreatment and enzymatic hydrolysis and co-conducted cost analysis. EK co-conducted the pretreatment and enzymatic hydrolysis experiments. AA co-conducted cost analysis. MF, MZ ND did the fermentation work. MH originated and managed the first biomass wet and dry milling research program at NREL, which provided the foundation and guidance for this work. DJ contributed research in deacetylation and mechanical refining. MT and RE led and coordinated the overall project. All authors have read and approved the final manuscript.

## Authors’ information

Dr. Ling Tao received her PhD degree in chemical engineering from University of Massachusetts at Amherst. She is now a senior process engineer in National Renewable Energy Lab. Her primary research interest is in process development and cost analysis of biofuel productions.
